# Marker-assisted backcross breeding to develop wheat lines with stable yellow rust resistance without yield penalties

**DOI:** 10.3389/fpls.2026.1895658

**Published:** 2026-09-08

**Authors:** Chandra Nath Mishra, Satish Kumar, Disha Kamboj, Garima Singroha, Nisha Kataria, Davinder Sharma, Arun Thakur, Rekha Malik, Pramod Prasad, Om Prakash Gangwar, Ratan Tiwari

**Affiliations:** Indian Council of Agricultural Research (ICAR)-Indian Institute of Wheat and Barley Research, Karnal, India

**Keywords:** adult plant resistance, marker assisted backcross breeding, seedling resistance, varietal development, wheat, yellow rust

## Abstract

Wheat production faces severe yield threats globally and regionally in India from rust diseases caused by *Puccinia* species. Genetic resistance breeding is the most sustainable approach to mitigate these losses, but traditional phenotypic selection is often constrained by environmental fluctuations and rapid pathogen evolution. Here we utilized marker-assisted backcross breeding (MABB) to introgress broad-spectrum yellow rust resistance genes, *Yr10* and *Yr15*, from the donor genetic stock PBW703 into the elite, high-yielding wheat cultivar DBW88 to develop two high-yielding rust-resistant genotypes DBW476 and DBW477. Foreground selection was executed using gene-linked molecular markers PSP3000 (*Yr10*) and Xbarc8 (*Yr15*). Background selection with 107 polymorphic simple sequence repeat (SSR) markers accelerated recurrent parent genome recovery up to 95% within just two backcross generations, significantly outperforming conventional breeding timelines. Seedling resistance tests and adult plant resistance evaluations confirmed that the introgressed lines exhibited a high degree of immune response and comprehensive resistance against virulent *Puccinia striiformis* pathotypes, including 238S119 and 110S119. Multi-year agronomic testing across multiple environments demonstrated that the improved near-isogenic lines achieved equal or superior grain yield performance compared to their recurrent parents and commercial checks. These findings indicate successful gene introgression without any associated linkage drag or yield penalties. These advanced, stable lines were nominated for multi-location varietal trials, presenting a strategically superior asset for sustainable rust management and food security in the rust-prone environments of India.

## Introduction

Wheat stands as a vital staple food for the world, especially for densely populated regions like India, China, and the European Union. In 2024–2025, China (140.1 million tonnes) followed by India (117.92 million tonnes) were the top two producers of wheat. By 2050, India’s population is estimated to grow to 1.7 billion people, and India’s wheat needs are estimated to be 125 metric tonnes of raw wheat. Wheat rust fungus *Puccinia* sp. causes severe yield losses in susceptible cultivars. According to FAO and CIMMYT 2014–2020, global loss of wheat yield was estimated at approximately 15 million tonnes due to rust infection amounting to 3–5 billion US dollars per year (Bhavani et al., 2022). In India, Punjab and Haryana bear the brunt of rust infection during disease infection years, recording annual yield losses of 30%–40%. Depending on variety and disease severity, documented yield losses have reached up to 70%, even 100% in extreme cases (Srinivas et al., 2021). In northwestern India, stripe rust yield loss has been recorded to vary from 4.2% to 68.8% in different wheat varieties under epidemic pressure (Singh, 2022). In an intensive study done on 19,460 wheat germplasm accessions by Kumar et al. (2017), it was found out that, in some accessions, yield loss measured up to 80%. Historical outbreak records globally show that rusts have caused serious losses and food security threats in earlier periods. Dr. KC Mehta, a pioneering plant pathologist, was first to document reports of yellow rust in India during the 1920s and 1930s (Nagarajan and Joshi, 1985).

Wheat stripe rust, leaf rust, and stem rust caused by *P. striiformis* f. sp. *tritici* (*Pst*), *P. triticina* (*Pt*), and *Puccinia graminis* f. sp. *tritici* (*Pgt*), respectively, cause significant yield losses in epidemic situations and major constraints to wheat production (Saari and Prescott, 1985; Bolton et al., 2008; Kosgey et al., 2021). Epidemic situations like these are often related to three main factors, i.e., host susceptibility, pathogen inoculum load, and congenial environment. Resistance breeding is the most environmentally and economically feasible approach to reduce losses due to these epidemics (Duveiller et al., 2007; Zhang et al., 2021).

In India, out of the total wheat cultivated area, approximately 10 million hectares is prone to stripe rust, and approximately 7.0 million hectares is prone to be affected by stem rust. Due to prevailing environmental conditions, leaf rust is a threat to wheat production across all the wheat-growing regions (Bhardwaj et al., 2019). Bhardwaj et al. (2016) have estimated a loss of approximately 39,200 million rupees due to rusts in India, and out of this approximately 10,000 million rupees is due to stripe rust alone. So far, 145 rust pathotypes have been identified in India (Bhardwaj et al., 2019). To date, 83 stripe rust (*Yr*) resistance genes have been permanently characterized and catalogued in wheat (McIntosh et al., 2017). All stage resistance genes or seedling resistance genes are vertical in nature and are characterized by a strong immune response which restricts disease development (Jamil et al., 2020). The race-specific gene *Yr10* (Yuan et al., 2018) is effective against the prevalent races of respective rust diseases. *Yr15*, on the other hand, which is derived from *T. dicoccoides* provides resistance against many prevalent stripe rust pathotypes. Exhibiting broad spectrum resistance compared to *Yr10*, it has remained effective against a large number of races globally for over two decades (Aboukhaddour et al., 2018).

Backcrossing (BC) describes a plant breeding procedure used to incorporate one or several genes into an adapted or elite variety. The BC method is a form of recurrent hybridization by which a superior characteristic is added to an otherwise desirable genetic background. In this method, the breeder has considerable control over the genetic variation in the segregating population in which the selections are to be made. Marker-assisted backcross breeding has now been employed to introgress the resistance genes in high-yielding background. Robust molecular markers are now available for several commercially important rust resistance genes (Bariana et al., 2007; Kamboj et al., 2020). The goal of the backcross program is to recover a pure line or inbred that will contain the novel allele/gene and be as good as the recurrent parent for all other important traits. This method has been extensively used for transferring alleles for novel traits into elite germplasm. In our study, we have improved the commercial wheat variety DBW88 (cultivated in North Western India) to develop two high-yielding rust-resistant genotypes, DBW476 and DBW477. The new backcross-derived lines DBW476 (DBW88+*Yr10*+Yr15) and DBW477 (DBW88+*Yr10*+*Yr*15) have shown equal or better agronomic performance with a high degree of resistance for yellow rust.

## Materials and methods

### Plant material and crossing program

Wheat cultivar DBW88 was used as the recipient in a breeding program. DBW88 (KAUZ//ALTAR84/AOS/3MILAN/KAUZ/4/HUITES) is a high-yielding variety released for cultivation in the Northern Western Plains Zone of India for irrigated timely sown conditions during 2014 (Tiwari et al., 2014). The genetic stock PBW703, having *Yr10* and *Yr15* (Bains et al., 2017), was used as a donor for yellow rust resistance.

**Figure 1:**
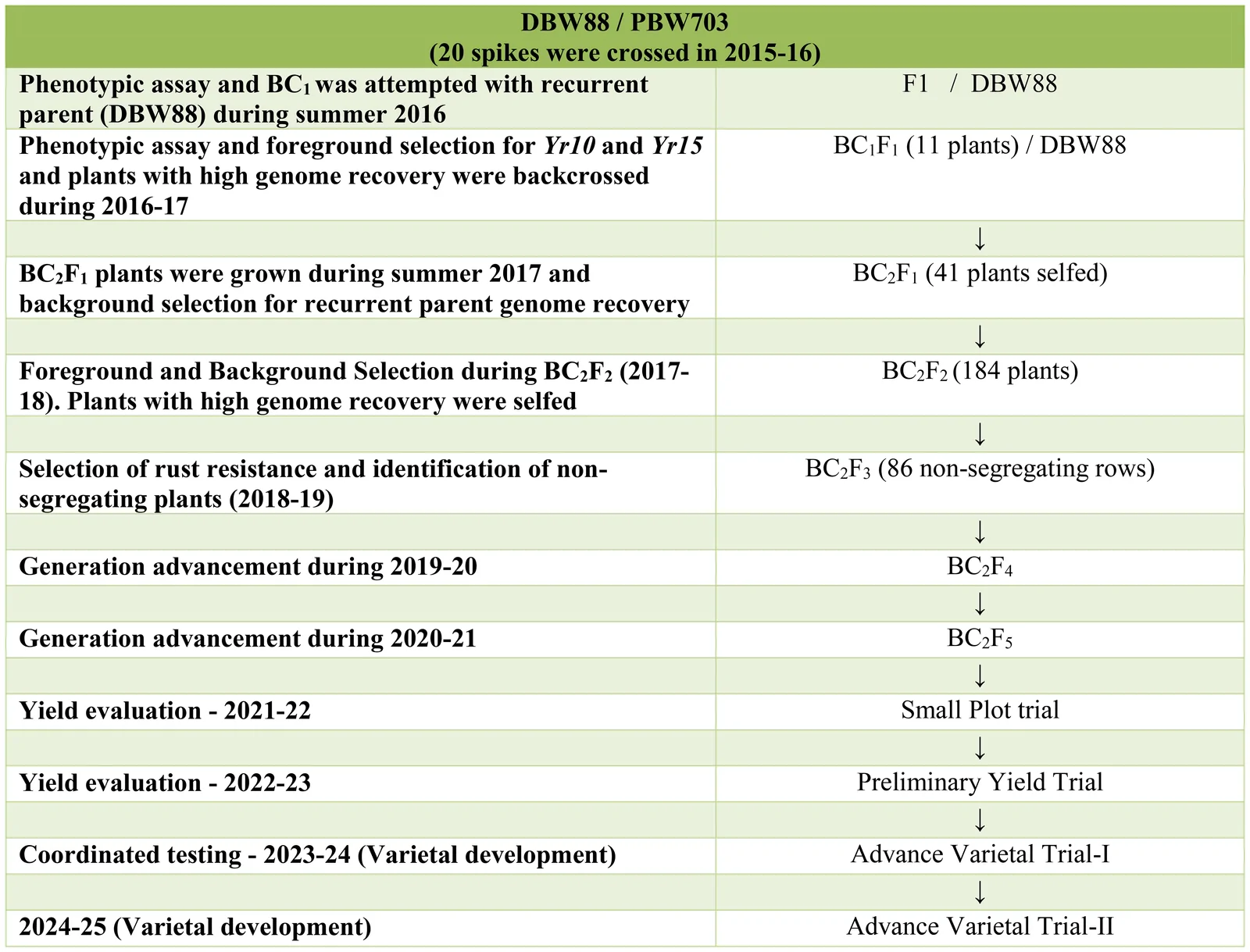
Schematic diagram depicting the marker-assisted transfer of *Yr10* and *Yr15*.

Marker-assisted backcross approach was followed to develop the near-isogenic lines (**Figure 1**). A cross was attempted between DBW88 and PBW703 during the crop season 2015–2016. F_1_s were crossed with the respective recurrent parent to generate BC_1_F_1_. The marker-selected plants were again backcrossed to the recurrent parent to generate BC_2_F_1_. In BC_2_F_2_ generation, the plants with higher genome recovery based on background selection were selfed, and non-segregating lines in BC_2_F_3_ generation were identified for evaluation during 2018–2019. The NILs were subjected to seedling resistance test for the predominant races of all the three rusts. The developed lines were evaluated for yield and agronomic traits, including disease resistance during 2019–2020 and 2020–2021. Out of these, two superior-yielding rust-resistant lines, DBW476 and DBW477, were contributed to the varietal development program and evaluated across multiple locations during 2023–2024 and 2024–2025.

### Genotyping

Fresh leaves were collected from 4- to 6-week-old plants of parental lines and backcross progenies, and the genomic DNA was isolated following the modified CTAB method of Saghai-Maroof et al. (1984).

Markers linked to rust-resistant genes were validated, where one marker linked to *Yr10* and one marker linked to the rust-resistant gene *Yr15* were used for foreground selection based on the published results (**Table 1**). The primers were synthesized by Eurofins Genomics India Pvt Ltd., Bengaluru, India. To identify polymorphic SSR markers suited for background selection, 800 established primer pairs were tested between donor and recipient parents, and the polymorphic markers were used for background selection. The primer sequences for the primer pairs were obtained from Grain-Genes 2.0: A database for Triticaceae and Avena (https://graingenes.org/GG3/frontpage?feature_acc=2723601&page=9).

**Table 1:** Molecular markers applied for the foreground selection of rust genes.

Gene	Linked markers	Size of fragment (bp)	Sequence of primers	Reference
*Yr10*	PSP3000	300	F - GCAGACCTGTGTCATTGGTC R - GATATAGTGGCAGCAGGATACG	Yuan et al. (2018)
*Yr*15	Xbarc8	260	F - GCGGGAATCATGCATAGGAAAACAGAA R - GCGGGGGCGAAACATACACATAAAAACA	Yaniv et al. (2015)

### Agronomic evaluation of backcross progenies

The field evaluation of marker-assisted-derived backcross lines (DBW476 and DBW477) was carried out along with recurrent parent DBW88 and commercial checks DBW187 and DBW222 at ICAR-Indian Institute of Wheat and Barley Research, Karnal, India, for 2 years (2019–2020 and 2020–2021). During 2019–2020, in BC_2_F_4_ generation, the lines were planted in a plot size of 3.2 m^2^, and data were recorded for yield and yield attributes. During 2020–2021, in BC_2_F_5_ generation, the advance lines were planted in a plot size of 7.2 m^2^, and data on days to 50% heading, days to physiological maturity, plant height, number of tillers per meter length, plant height, spike length, flag leaf length and width, grains per spike, thousand grains weight, harvest index, NDVI, canopy temperature, and grain yield per plant were recorded. During 2021–2022, the selected lines were advanced for small plot trials, followed by preliminary yield trials in 2022–2023 and then advanced varietal trial-I (AVT-I) in 2023–2024.

### Phenotyping for rust resistance

Strict screening was carried out for yellow rust and leaf rust during the development of the material at Karnal; however, to confirm the resistance, the seedling resistance test (SRT) against the predominant races of yellow rust (238S119 and 110S119) was carried out at the ICAR-IIWBR Regional Station, Shimla, India, during 2019–2020. Aluminium trays containing a sterilized mixture of fine loam and farmyard manure (3:1) were used for raising seedlings in spore-proof chambers at 20 + 2°C, 50%–70% relative humidity, and 12 h of day light. One-week old seedlings were inoculated with 10 mg of spores of an individual pathotype suspended in 1 mL of light-grade mineral oil (Soltrol 170; Chevron Phillips Chemicals Asia Pvt. Ltd., Singapore). The infection types (IT) were recorded at 16 days post-inoculation using standard procedure (Nayar et al., 1997).

## Results

### Marker-assisted transfer

**Figure 2:**
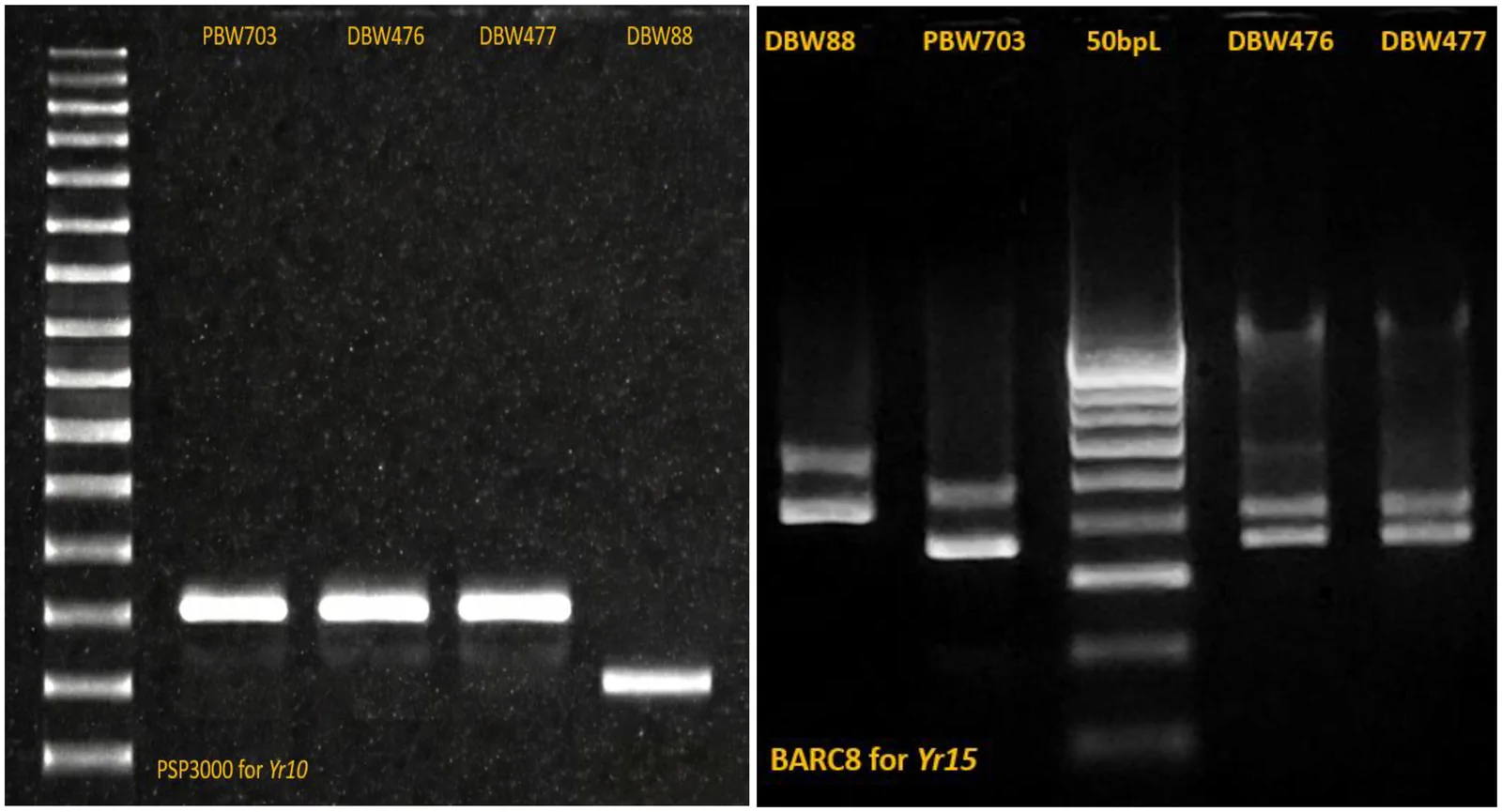
Marker profile of the genes *Yr10* and *Yr15*.

PBW703 was crossed with DBW88 to produce F_1_ hybrids which were further backcrossed with the recurrent parent (recipient variety). Phenotypic assay and marker-assisted selection (**Figure 2**) with Xbarc8 for *Yr15* and PSP3000 for *Yr10* were carried out in BC_1_F_1_ generation in the cross, and 11 positive plants were identified in PBW703/DBW88. These were backcrossed with the recurrent parent to produce the BC_2_F_1_ generation, where 41 were identified on the basis of foreground and phenotypic evaluation. These plants were selfed to produce BC_2_F_2_. The detailed transfer is illustrated in **Figure 1**.

Marker PSP3000 was used to track the transfer of the *Yr10* gene from PBW703 to DBW88. On the basis of field evaluation and marker data, 41 plants of DBW88/PBW703 were selected and selfed to produce the BC_2_F_2_ generation.

### Background selection

**Figure 3:**
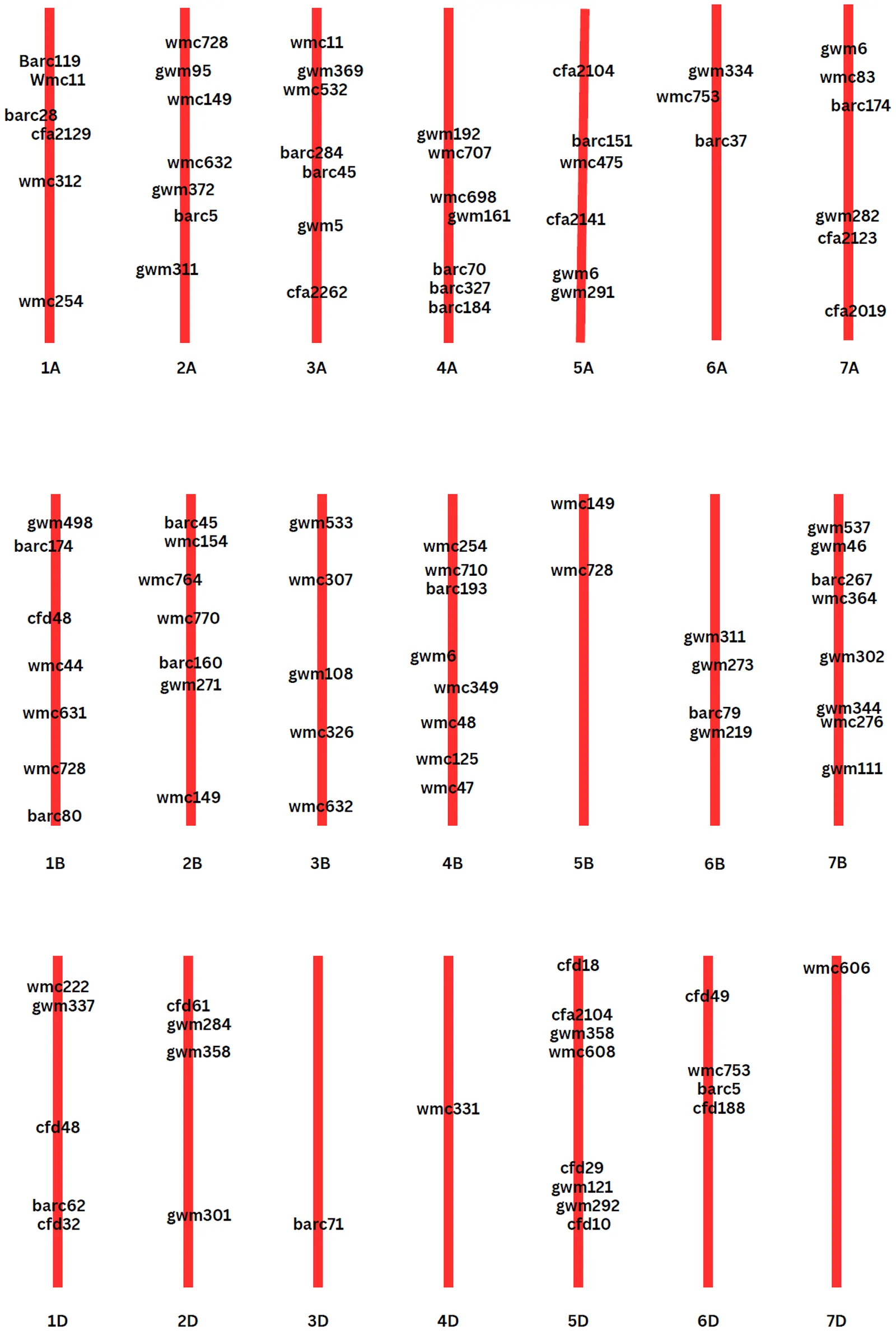
Map location of informative SSR used for background selection.

**Figure 4:**
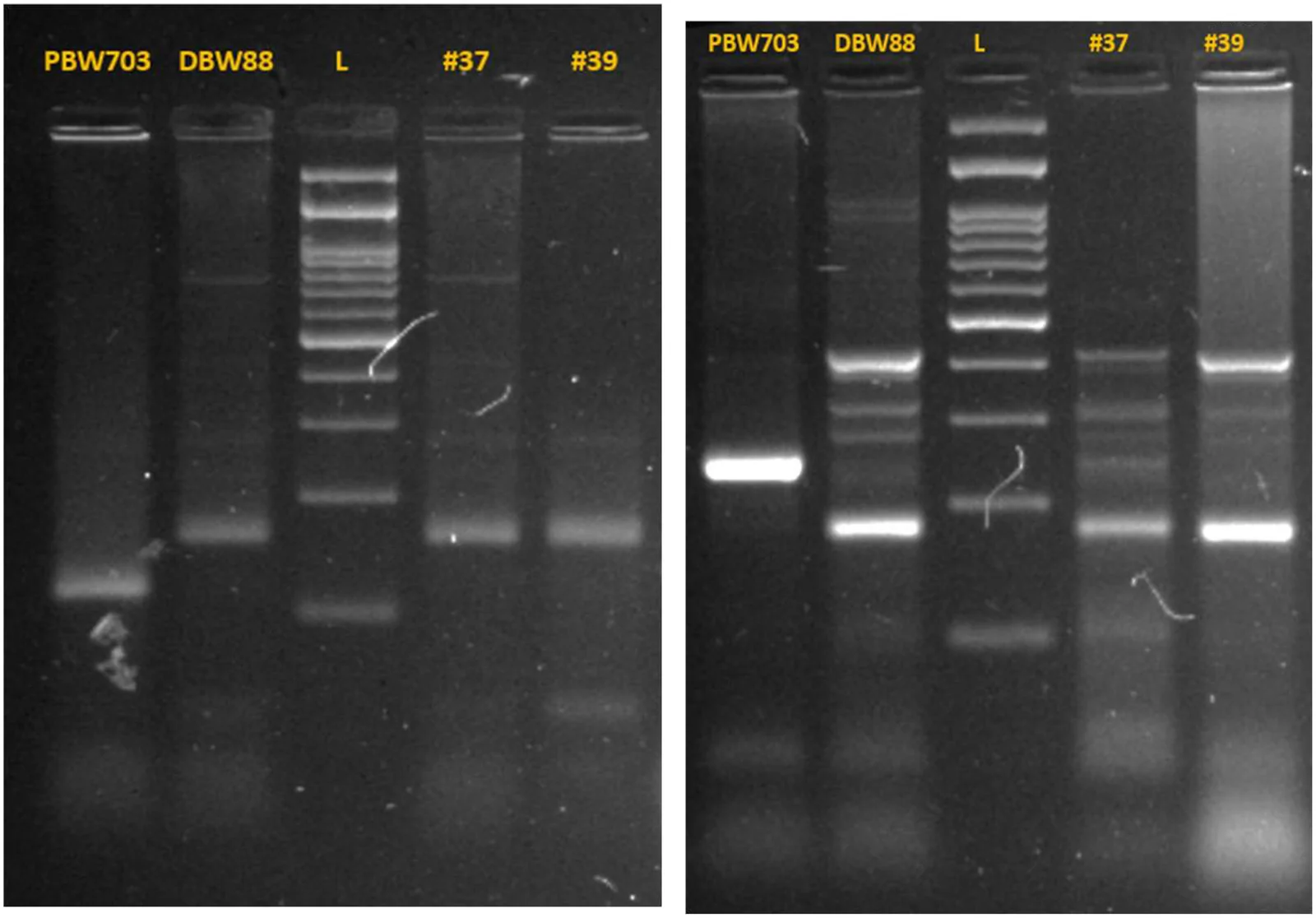
Representative gel images of background selection with markers gwm311 (left) and gwm219 (right). #37 represents DBW476 and #39 represents DBW477.

From a set of approximately 400 markers, 107 were identified as polymorphic between the parents used in the cross combinations. Their map location on different chromosomes is presented in **Figure 3**. SSR analysis in BC_2_F_2_ generation carried out in different crosses helped in recovering the genome of the recurrent parent up to 95% in different cross combinations. **Figure 4** shows the confirmation of resistant genes *Yr10* and *Y15* in the introgressed lines DBW476 and DBW477.

### Confirmation of rust-resistant lines through SRT analysis

The non-segregating lines for agronomic traits were identified in each cross combination in BC_2_F_3_ during crop season 2018–2019. The promising lines having better agronomic traits were subjected to a seedling resistance test during the summer season of 2019, and a part of the result along with the recurrent parent is presented in the table below. The transfer of *Yr15* to DBW88 improved the resistance of these cultivars against the most virulent races 238S119 and 110S119. This, combined with *Yr10* in the same background, conferred resistance against most prevalent races of yellow rust as shown in **Table 2a**. The two selected, *viz*., #37 and #39, were subjected to seedling and APR response in AVT 2023–2024. DBW88 had a high ACI, indicating poor APR along with high severity (**Table 2b**), whereas DBW477 showed low ACI scores but was still on the susceptible spectrum, suggesting slow progression of rust, indicating quantitative APR. DBW476 had the lowest ACI score and lowest susceptibility and showed very slow rusting, indicating APR at work.

**Table 2a:** Seedling response of the selected superior lines and parents.

Pathotype	Seedling response to yellow rust														
	46S119	110S119	238S119	78S84	110S84	P	T	79S4	79S68	111S68	K	N	6S0	7S0	14S64
DBW88	S	S	S	R	MS	R	R	R	R	R	R	–	R	R	R
PBW703	R	R	R	R	R	R	R	R	R	R	R	R	R	R	R
DBW477	R	R	R	R	R	R	R	R	R	R	R	R	R	R	R
DBW476	R	R	R	R	R	R	R	R	R	R	R	R	R	R	R

**Table 2b:** Adult plant resistance response of the selected superior lines and parents.

Adult plant resistance for yellow rust		
Genotypes	ACI	HS
DBW88	27.6	60S
PBW703	2.5	20MS
DBW477	3.8	20S
DBW476	2.2	10S

ACI, average coefficient of infection; HS, highest score.

### Agronomic evaluation of backcross-derived lines—2020–2021

A total of 137 promising lines were subjected to agronomic evaluation during the 2019–2020 crop season in the BC_2_F_4_ generation. The promising lines in each combination were identified on the basis of yield and yield-attributing traits. Furthermore, 47 selected lines from BC_2_F_5_ were evaluated in simple lattice design against two check varieties, DBW187 and DBW222. A comparatively high variation was observed for flag leaf width (19.82%), tillers per meter (18.81%), grain yield (15.18%), and grains per spike (13.54%). Harvest index and plant height contributed positively, whereas spike length, flag leaf length, and width contributed negatively for grain yield.

Two promising lines having *Yr10*+*Yr15*, *viz*., #37 and #39, were selected for the large plot testing against the commercial check varieties. Small plot trials were conducted in 2020–2021, followed by PYT in 2022–2023 and AVT in 2023–2024 (**Tables 3a**, **b**). The two selected lines in this cross were similar in plant height, spike length, and number of grains per spike; however, an improvement in 1,000-grains weight and spike yield leading to a total increase in yield per plot in the improved lines of DBW88 was due to improvement in yellow rust resistance.

**Table 3a:** Yield performance of the introgressed lines and parents during 2023–2024.

Genotypes	Yield (q/ha) 2023–2024							
	Delhi	J&K	Punjab	Haryana	Rajasthan	U.P.	Uttarakhand	Zonal
DBW88	76	57.9	57.8	57.4	67.3	61.1	61.8	62.0
DBW477	61.9	61.0	64.1	62.7	65.2	59.0	59.1	61.9
DBW476	62.4	55.9	63.8	55.3	63.3	58.3	61.5	60.0

**Table 3b:** Agronomic performance 2023–2024.

Yield related traits	DBW88		DBW476		DBW477	
	Mean	Range	Mean	Range	Mean	Range
	2023–2024	2023–2024	2023–2024	2023–2024	2023–2024	2023–2024
Days to heading	100	79–111	100	78–109	100	134–150
Days to maturity	146	88–112	145	135–158	144	134–150
Plant height	104	88–112	105	94–114	103	90–110
1,000-grain weight	39	30–45	40	32–50	40	30–51

## Discussion

Genetic resistance remains the most cost-effective, environmentally safe, and farmer-friendly approach for minimizing yield losses caused by rust diseases in wheat (Kolmer, 1996). Over the years, a large number of rust resistance genes have been identified from diverse genetic sources and successfully incorporated into cultivated wheat backgrounds to enhance resistance levels (Trethowan et al., 2018). However, the effectiveness of resistance based solely on phenotypic selection is often limited by environmental variation, race specificity of the pathogen, and difficulties in reliable disease expression, particularly under fluctuating field conditions. To overcome these limitations, marker-assisted selection has emerged as a powerful supplementary tool to conventional breeding and has gained widespread acceptance during the last two decades (Dubcovsky, 2004; Gupta et al., 2009; Kamboj et al., 2020).

Modern resistance breeding is increasingly shaped by the ongoing coevolutionary arms race between wheat and rust pathogens, where breeders continuously deploy new resistance strategies while *Puccinia* species evolve to overcome them. Contemporary breeding approaches therefore emphasize strategic deployment of major resistance (R) genes, quantitative adult plant resistance (APR) genes, pyramided gene combinations, genomic-selection-assisted breeding lines, and gene-edited variants to achieve durable resistance (Kumar et al., 2019). In contrast, rust pathogens respond through mutation, somatic recombination, genetic exchange, and long-distance migration, enabling rapid adaptation in regions where susceptible cultivars dominate (Singh et al., 2025). This dynamic interaction underscores the necessity of integrating multiple resistance mechanisms within elite cultivars rather than relying on single resistance genes.

The overall efficiency of a marker-assisted backcross breeding (MABB) program is largely dependent on the availability of tightly linked molecular markers for foreground selection and the effective assessment of recurrent parent genome recovery using genome-wide polymorphic markers (Muthu et al., 2020). Precise foreground selection ensures the accurate transfer of target genes, while background selection accelerates the recovery of the recurrent parent genotype, thereby reducing linkage drag and shortening the breeding cycle. Numerous successful studies have demonstrated the effectiveness of MABB for introgression and pyramiding of major resistance genes and QTLs in wheat (Barloy et al., 2007; Gupta et al., 2010; Kumar et al., 2010; Tester and Langridge, 2010; Malik et al., 2015; Shah et al., 2017; Sharma et al., 2021).

In the present study, the successful introgression of *Yr10* and *Yr15* from the donor parent PBW703 into the genetic background of DBW88 was achieved within two backcross generations, which is considerably faster than the conventional backcrossing approach. This accelerated recovery was made possible through the use of gene-linked molecular markers, which enabled the precise tracking of the resistance genes at each generation. The use of such tightly linked markers minimizes the risk of false selection and ensures faithful transmission of the desired alleles. The results reaffirm that marker-assisted backcross breeding not only enhances selection efficiency but also substantially reduces time, resources, and field evaluations required for resistance breeding, making it an indispensable approach for modern wheat improvement programs.

Here we introduced two rust resistance genes, *Yr10* and *Yr15*, for improving stripe rust resistance in the background of the popular wheat variety DBW88 through a marker-assisted backcross breeding (MABB) approach and evaluated their performance against virulent pathotypes. *Yr15* is known to confer high-level and broad-spectrum resistance to stripe rust across diverse genetic backgrounds, while *Yr10* provides effective race-specific resistance under Indian conditions (McIntosh et al., 2017; Chen, 2013). The improved lines were also evaluated agronomically against commercial cultivars to identify superior lines suitable for commercial cultivation. The best-performing lines were advanced from small plot trials during 2021–2022 to AVT-I in 2023–2024, indicating their agronomic competitiveness and stability under field conditions.

Out of approximately 400 SSR markers screened, 107 markers showed polymorphism between the parents used in the cross combination, and their chromosomal distribution is presented in **Figure 3**. These polymorphic markers were employed for genome-wide background selection to estimate recurrent parent genome recovery in the BC_2_F_2_ generation. The SSR-based analysis revealed recurrent parent genome recovery of up to 95% across different plants, which is markedly higher than the theoretical expectation of ~87.5% at the BC_2_ stage under conventional backcrossing. Comparable levels of accelerated genome recovery have been reported in wheat MABB programs, where 92%–97% recurrent parent genome recovery was achieved by BC_2_–BC_3_ generations through marker-assisted background selection (Randhawa et al., 2009; Malik et al., 2015; Sharma et al., 2021), further validating the efficiency of the approach adopted in the present study.

Gene-linked molecular markers assist the selection process in plant breeding programs for traits that are phenotypically complex, environmentally influenced, or difficult to score reliably under field conditions (Koebner and Summers, 2003). Rust resistance, particularly at adult plant stages, is highly influenced by environmental factors and pathogen variability; therefore, marker-assisted selection provides a reliable and precise method for tracking resistance genes across generations (Singh et al., 2016a).

In a conventional backcrossing program, the general practice is to advance material up to BC_4_ generation based on theoretical expectations that approximately 87.5% of the recurrent parent genome is recovered at this stage (Allard, 1999). However, the integration of molecular markers with phenotypic selection substantially accelerates recurrent parent genome recovery and reduces the number of backcross generations required (Hospital, 2001). In the present study, we observed individual genetic recovery up to 95% by BC_2_ generation, clearly demonstrating the efficiency of background selection in MABB.

Comparable results have been reported earlier, where genetic recovery up to 97% was achieved during introgression of *Yr15* in wheat through marker-assisted backcrossing (Randhawa et al., 2009). Similarly, accelerated recurrent parent genome recovery of approximately 95% by BC_2_ generation has been documented in rice using molecular-marker-based background selection (Neeraja et al., 2007). These findings collectively confirm that marker-assisted backcross breeding is an effective strategy for the rapid improvement of elite cultivars while retaining their desirable agronomic traits.

The agronomic performance of the improved DBW88-derived lines indicated that introgression of *Yr10* and *Yr15* did not impose any yield penalty or adverse effect on key agronomic traits, suggesting minimal linkage drag from the donor parents. This is a critical consideration for farmer adoption, as resistance genes must be incorporated without compromising yield potential or adaptation (Singh et al., 2016b).

From a resistance durability perspective, pyramiding of major rust resistance genes is considered a strategically superior approach compared to deployment of single genes, as it reduces the probability of resistance breakdown due to rapid pathogen evolution (McIntosh et al., 2017). The successful pyramiding of *Yr10* and *Yr15* in DBW88 thus represents a valuable contribution toward sustainable rust resistance breeding in wheat, particularly for the rust-prone environments of the Northern Western Plains Zone of India.

## Conclusion

The improved lines developed in the genetic background of DBW88 carrying enhanced rust resistance were subsequently nominated for multi-environment evaluation with the objective of assessing their stability, adaptability, and suitability for large-scale cultivation. Advancement of these lines to multi-location testing represents a critical step toward the deployment of the introgressed resistance genes under diverse agro-climatic conditions, ensuring their effectiveness against varying rust pathotypes. This strategy aimed to combine major gene resistance with additional resistance components present in the donor lines, thereby enhancing durability and broadening the spectrum of protection. The integration of marker-assisted selection with multi-environment phenotypic evaluation is expected to accelerate the development of high-yielding, rust-resistant wheat cultivars, contributing to sustainable wheat production and long-term disease management.

## Data Availability

The original contributions presented in the study are included in the article, further inquiries can be directed to the corresponding author.
